# Detection of Epileptic Seizures Using Phase–Amplitude Coupling in Intracranial Electroencephalography

**DOI:** 10.1038/srep25422

**Published:** 2016-05-05

**Authors:** Kohtaroh Edakawa, Takufumi Yanagisawa, Haruhiko Kishima, Ryohei Fukuma, Satoru Oshino, Hui Ming Khoo, Maki Kobayashi, Masataka Tanaka, Toshiki Yoshimine

**Affiliations:** 1 Osaka University Graduate School of Medicine, Department of Neurosurgery, Suita 565-0871, Osaka, Japan; 2 Osaka University Hospital Epilepsy Center, Suita 565-0871, Osaka, Japan; 3 ATR Computational Neuroscience Laboratories, Department of Neuroinformatics, Seika-cho 619-0288, Kyoto, Japan; 4 JST PRESTO, Suita 565-0871, Osaka, Japan; 5 Osaka University Graduate School of Medicine, Division of Functional Diagnostic Science, Suita 565-0871, Osaka, Japan

## Abstract

Seizure detection using intracranial electroencephalography (iEEG) contributes to improved treatment of patients with refractory epilepsy. For that purpose, a feature of iEEG to characterize the ictal state with high specificity and sensitivity is necessary. We evaluated the use of phase–amplitude coupling (PAC) of iEEG signals over a period of 24 h to detect the ictal and interictal states. PAC was estimated by using a synchronisation index (SI) for iEEG signals from seven patients with refractory temporal lobe epilepsy. iEEG signals of the ictal state was characterised by a strong PAC between the phase of β and the amplitude of high γ. Furthermore, using SI values, the ictal state was successfully detected with significantly higher accuracy than by using the amplitude of high γ alone. In conclusion, PAC accurately distinguished the ictal state from the interictal state.

The detection of epileptic seizures using intracranial electroencephalography (iEEG) is crucial for the treatment of epilepsy and revealing its pathophysiology. Intracranial electrodes have enabled the generation of a higher signal-to-noise ratio and a wider frequency band than that obtained by scalp electroencephalography (EEG). It has been reported that iEEG is useful for surgical decision-making regarding patients with magnetic resonance imaging (MRI)-negative temporal lobe epilepsy (TLE)^1^. Some investigators have attempted to develop systems for the automatic detection of epileptic seizures with iEEG, which will be beneficial for patients with epilepsy^2^,^3^,^4^,^5^. Previous studies revealed several characteristic features of iEEG during a seizure attack: rhythmic waveforms in the δ to low-γ frequency range^6^, a high frequency component (higher than the γ band)^7^,^8^,^9^, and a very low-frequency component (e.g., DC wave)^10^. Although these features are observed during a seizure, they are also observed during the interictal state, thereby occasionally causing false-positive detection of a seizure attack.

Recently, cross-frequency coupling has been identified as a physiologically relevant activity observed in various areas of the human brain that varies in a task-relevant manner^11^,^12^. For example, coupling between the θ phase and high γ amplitude is observed in the human temporal lobe and varies depending on some cognitive tasks^12^,^13^. Similarly, coupling between the α phase or β phase and high γ amplitude was shown in the human sensorimotor cortex and varies with sensory, motor, and cognitive tasks^14^.

Moreover, cross-frequency coupling of ictal iEEG was shown to characterise seizures successfully^15^,^16^,^17^. In particular, coupling between the low-frequency phase and high γ amplitude (phase–amplitude coupling [PAC]) was more useful for the localisation of an epileptic focus than the high γ amplitude alone^15^. However, it is unclear whether the ictal state can be distinguished from the interictal state by using only the strength of PAC of the higher frequency amplitude and the lower-frequency phase. Here, we compared PAC during the ictal and interictal states. We also evaluated whether PAC can detect the ictal state and, specifically, the interictal state in patients with MRI-negative TLE.

## Methods

### Subjects

Seventeen patients were implanted with intracranial electrodes at Osaka University Hospital from January 2013 to March 2015. Among these patients, we selected 7 (5 males and 2 females; Table 1) who met all the following inclusion criteria: (i) 16 years of age or older; (ii) a diagnosis of refractory TLE; and (iii) no abnormal lesion on 3 Tesla MRI scans. TLE was diagnosed based on semiology, video EEG, magnetoencephalography (MEG), single-photon emission computed tomography (SPECT), and fluorine-18 fluorodeoxyglucose positron emission tomography (FDG-PET). Here, we focused on the analysis of iEEG of patients with MRI-negative TLE, because most of the TLE patients who underwent subdural electrode implantation in our hospital had no abnormal lesion on MRI. In other words, iEEG analysis was clinically required for these patients. Only one patient had TLE with MRI-defined hippocampal sclerosis during the study period. Therefore, we only selected patients with MRI-negative TLE for two reasons: (1) these patients will potentially need iEEG analysis and (2) to make the patient population of this study uniform. All patients underwent video monitoring of epileptic attacks with iEEG recordings for 7–14 days after implantation of the electrodes. The dosage of antiepileptic drugs was reduced in some patients according to the guidelines for the non-invasive scalp video EEG. This study adhered to the Declaration of Helsinki and was performed in accordance with protocols approved by the Ethics Committee of the Osaka University Clinical Trial Centre (No. 14353, UMINID 17900). Each patient was informed of the purpose and possible consequences of this study, and written informed consent was obtained.

### Electrode implantation and iEEG acquisition

We implanted several types of electrodes in the subdural space during conventional craniotomy. The electrodes were planar-surface platinum grid electrodes (3 × 5, 4 × 5, or 5 × 6 array; Unique Medical, Tokyo, Japan) and 4–12 depth electrodes (Unique Medical). The contacts had a diameter of 3 mm and an intercontact distance of 7 or 10 mm centre to centre. Locations of the implanted electrodes were determined by two epileptologists based on seizure semiology and interictal and ictal findings of scalp EEG, MEG, SPECT, FDG-PET, Wada test, enhanced MRI (3.0-T MRI scanner Signa; GE Medical Systems, Milwaukee, WI, USA), and computed tomography (CT) (Discovery CT750 HD; GE Healthcare, Waukesha, WI, USA, or Toshiba Aquilion; Toshiba Medical Systems, Tokyo, Japan). Co-registration between the postoperative CT and preoperative MRI scans was obtained to create a 3-dimensional brain rendering of the MRI volume overlaid with the co-registered CT volume for an initial estimate of the position of the electrodes using iPlan^®^ Cranial (BrainLAB, Munich, Germany).

iEEG signals were acquired using a 128-channel clinical video EEG 2000 system (Nihon Kohden, Inc., Tokyo, Japan) at 1000–10,000 Hz per channel and downsampled to 1000 Hz using a filter (eegfilt.m from the EEGLAB toolbox; MATLAB 2014a). For each patient, the epileptologists verified the implanted electrodes at the beginning of the iEEG recording and identified contacts with noisy signals due to technical problems. These contacts were excluded from the analysis. We also excluded segments during which the electrodes were disconnected due to patient transfer. All signals were referenced to the common average of all the contacts.

Seizure onset and seizure onset zone were identified by two epileptologists independently using video-iEEG based on the ictal findings. The ictal findings were defined as the initial iEEG changes characterized by sustained rhythmic discharges or repetitive spike-wave discharges that cannot be explained by state changes and that resulted in habitual seizure symptoms similar to those reported in previous studies^18^,^19^,^20^,^21^. The earlier of the two identified onsets was used as the seizure onset. The seizure onset zone was defined as the contacts where the earlier ictal iEEG changes were seen. The resection area was determined based on the iEEG findings and other pre-surgical evaluations.

We defined the ictal state as the period of 2 minutes after the onset of the seizure and the interictal state as the period of 24 hours before or after onset, except for 30 minutes before and after the seizure. For patient F, we only managed to acquire a total of 10 hours of iEEG signals at 1000–10,000 Hz (Table 1). Thus, for this patient, only 8 hours of interictal data were analysed after removing the 30 minutes of pre-ictal and post-ictal data of the two seizures from the total of 10 hours of recording.

### Calculation of the synchronisation index

A synchronisation index (SI) was used to measure the strength of coupling between the amplitude and phase based on the instantaneous phase and amplitude calculated from the Hilbert transformation^22^. The SI was defined as:


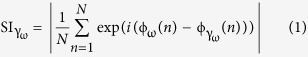


where N is the number of time points during each analysis section. 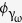, which is the phase of the low-frequency band (ω), was identified from the amplitude of the high frequency band (γ). The SI was calculated by finding the remainder between 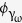 and the phase of ω. SI values were also calculated using phase-shuffled data to estimate SI values expected by chance (phase-shuffled SI). In the phase-shuffled data, the time series of the low-frequency phase was shuffled by permuting randomly partitioned segments^13^. The SI was calculated using MATLAB 2014a.

### Coupling of phase and amplitude for each combination of frequencies during a seizure

We evaluated the SI values of each contact for the ictal state and for the interictal state. For each second, the SI values were evaluated for the phase of 4-Hz bins from 2–6 to 38–42 Hz in 2-Hz intervals and for the amplitude of 70-Hz bins from 10–80 to 200–270 Hz in 10-Hz intervals. For each combination, t values were calculated by comparing the SI values to the phase-shuffled SI values using Student’s *t*-test with Bonferroni’s correction.

### SI during ictal and interictal states

The SI values between the phase of three lower-frequency bands (θ, 4–8 Hz; α, 8–13 Hz; β, 13–25 Hz) and the amplitude of the high γ band (80–150 Hz) and those between the phase θ band and the amplitude of the 10–80 Hz band were calculated for the ictal and interictal states. The 120-s signals starting from seizure onset for each patient were used for the ictal state, and the interictal state was defined as a period of 24 h without a seizure. The SI values were calculated for a 500-ms time window slide by 200-ms. The phase-shuffled SI values were then subtracted from the SI values to estimate the changes of the SI value in each time window (defined as “*SI”*).


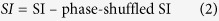


Here, we averaged 300 consecutive *SI* values, corresponding to 60 seconds, to examine the slow change of the *SI* value related to the ictal state, which usually lasts for more than 60 seconds, and to exclude the fast changes of the *SI* value related to the physiological alteration of PAC in the interictal state, which varies depending on the patient’s activities, such as motor, cognition, and mental tasks.

To examine the *SI* values during the ictal and interictal states of all patients, the *SI* values of each contact and each time window were normalised. In each contact of the patients, the *SI* values were *z*-scored using the mean and standard deviation (SD) of the *SI* values during the interictal state. In addition, the amplitude of high γ (80–150 Hz) was also calculated by the Hilbert transformation and was normalised in the same manner as the *SI* values. The distributions of the normalised *SI* values or high γ amplitudes during the ictal and interictal states were tested by the Mann-Whitney U test. To equalize the effect of individual differences on the distribution, the first seizure of each patient was used as the ictal state.

### Detection of a seizure using SI values

We evaluated how accurately the normalised *SI* values and the high γ amplitudes differentiated the ictal state from the interictal state. An ictal state was classified for each time window when the normalised *SI* value or high γ amplitude exceeded a certain threshold at a minimum of two contacts. By changing the threshold, we plotted the receiver-operating characteristic (ROC) curve of each normalised *SI* value and high γ amplitude to differentiate the ictal state from the interictal state. Using the area under the ROC curve (AUC), the accuracy detecting the ictal state was compared among the normalised *SI* values and high γ amplitudes (see methods by Hanley, J.A. & McNeil, B.J.^23^). Notably, only the first seizure of each patient was analysed to equalize the effect of individual differences on the results.

Sensitivity to detect the ictal state and the false detection rate (FDR) during the interictal state were evaluated using the normalised *SI* values or the high γ amplitude. The four types of normalised *SI* values and the high γ amplitude were evaluated as features to detect seizures. A seizure was detected when the feature values were classified as the ictal state at least once during 120 s after seizure onset. First, a threshold for each feature was determined as a minimal value to detect all first seizures of each patient. Then, the remaining seizures were tested with the threshold. The sensitivity of the detection was evaluated as the number of detected seizures divided by the number of remaining seizures. Moreover, the threshold was applied to the features during the interictal state to evaluate false detection. After an onset of detection, we skipped 30 seconds. The FDR was estimated as the number of seizures detected during the interictal state divided by the duration of the interictal state.

## Results

### PAC significantly increases during a seizure

**Figure 1:**
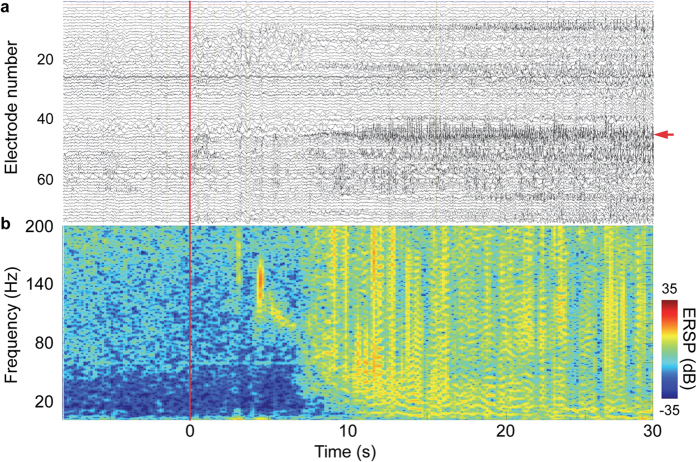
A sample of iEEG during a seizure in patient G. (**a**) The low-frequency (0.53–200 Hz) band pass–filtered iEEG signals of all implanted contacts are shown. Each raw trace corresponds to each contact. The vertical red line represents the seizure onset. The red arrow indicates contact no. 45 used to plot map (**b**). (**b**) Time-frequency map showing colour-coded event-related spectral perturbation (ERSP) for contact no. 45 at the approximate time of seizure onset.

**Figure 2:**
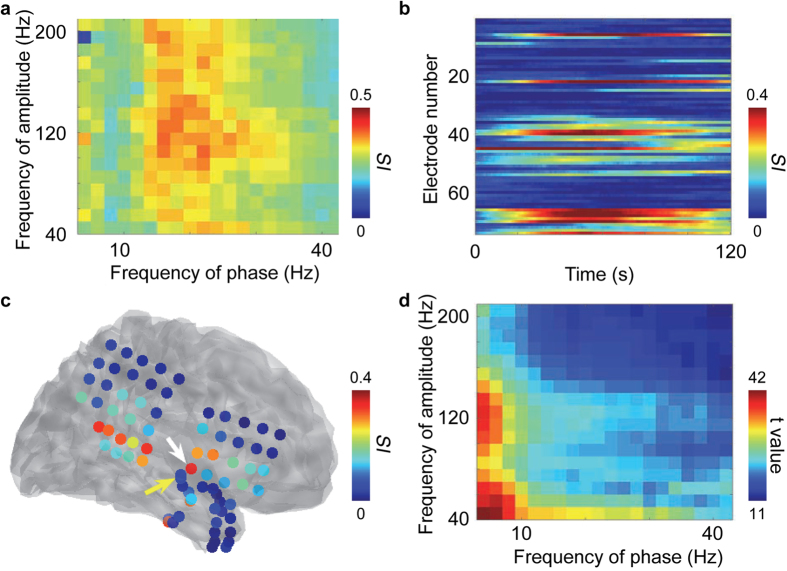
PAC of iEEG signals during an ictal state in patient G. (**a**) The averaged *SI* values during the ictal state are shown for all combinations of phase and amplitude frequencies. The ictal state was defined as 120 s from the onset of the first seizure. (**b**) The *SI* values of β-high γ were colour-coded for each contact over time during the ictal state. (**c**) The averaged *SI* of β-high γ values during the ictal state was colour-coded at the location of each contact in a normalised brain. The yellow arrowhead indicates the epileptogenic zone. The white arrowhead indicates the contact no. 45. (**d**) For each contact in all the patients, Student’s *t*-test was used to identify significant differences between the averaged SI values and the averaged phase-shuffled SI values during the ictal state. The *t*-values were colour-coded for each combination of phase and amplitude frequency.

A total of 21 seizures were recorded from 7 patients using video-iEEG (Table 1). All seizures were complex partial seizures with or without secondary generalisation. During the seizures, we observed several characteristic features of the iEEG signals. Figure 1a shows a representative example of the iEEG signals in a patient (patient G). Time-frequency analysis for contact no. 45 demonstrated an increase in high γ power after seizure onset, and the power varied over several seconds (Fig. 1b). The *SI* values between different frequencies of amplitudes and phases were evaluated for the same iEEG contact, as analysed in Fig. 1b. The *SI* values between the β phase and high γ amplitude (*SI* of β-high γ) were larger than *SI* values between the other combinations of frequency bands (Fig. 2a). Among all the contacts of the same patient (patient G), the *SI* value of β-high γ increased after seizure onset and was maximized at approximately 1 min after onset at several contacts (Fig. 2b). Moreover, when contacts were colour-coded according to the mean *SI* values of β-high γ during the seizure, high *SI* values were located around the temporal lobe, including the seizure onset zone (Fig. 2c). For all patients, the *SI* values increased during the ictal state on the temporal lobe. Moreover, the *SI* values of β-high γ were highest within the seizure onset zone identified by the epileptologists using waveforms of the iEEG signals for three of the patients (see Supplementary Fig. S1). For the first seizure of all the patients, the mean SI values and the mean phase-shuffled SI values of each contact were compared using the paired Student’s t-test (n = 613). High t values seen in the amplitude at 40–120 Hz at the phase of 4–30 Hz clearly demonstrated that the high γ amplitude was coupled with the phase of the θ or β band (Fig. 2d).

### Ictal PAC is significantly stronger than interictal PAC

**Figure 3:**
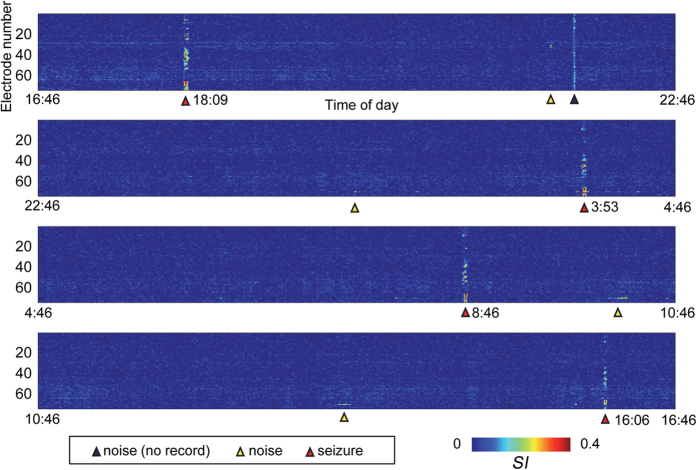
The *SI* values of β-high γ of each contact were colour-coded over a 24-h period in patient G. The seizures are indicated with a red arrowhead. The black arrowhead indicates the duration during which iEEG was disconnected. The white arrowhead indicates some artefacts from unknown origins.

Although the *SI* values varied during the interictal state, the *SI* values of the ictal state were larger than those of the interictal state. Figure 3 shows a sample of *SI* values for 24 h for patient G. The *SI* values of all the contacts varied between the interictal states depending on the patient’s behaviour. Nevertheless, the *SI* values of the ictal state were notably larger than those of the interictal state.

**Figure 4:**
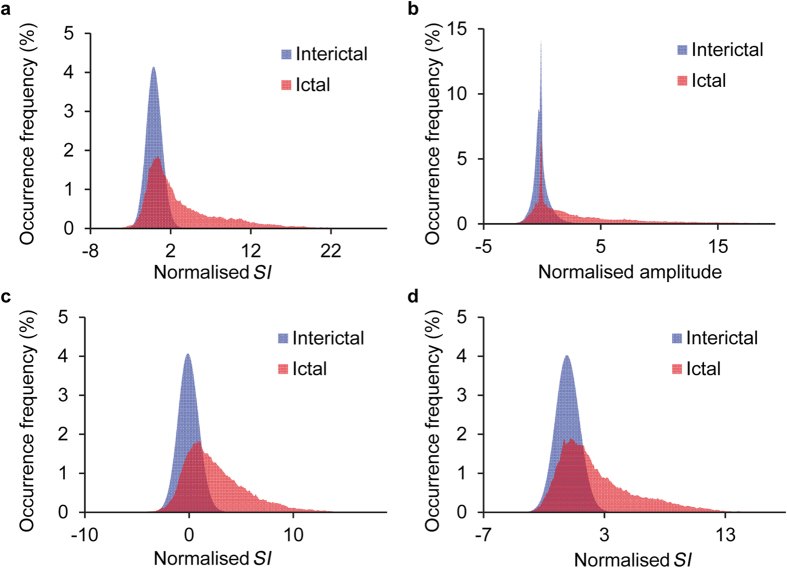
The distribution of normalised *SI* values and high γ amplitude during ictal and interictal states. (**a–d**) iEEG signals of each contact in all the patients were used to estimate (**a**) *SI* values of β-high γ, (**b**) high γ amplitude, (**c**) *SI* values of θ-high γ, and (**d**) *SI* values of α-high γ amplitude. Interictal state, blue; ictal state, red.

The frequency distribution of the normalised *SI* values was significantly different between the ictal and interictal states. The frequency distributions of the *SI* values or high γ amplitudes of each contact of all the patients were normalised by the mean and SD to compare between the ictal and interictal states. The normalised *SI* values of β-high γ increased with a long tail distribution during the ictal state compared to those during the interictal state (Fig. 4a). The two distributions were significantly different (*p* < 0.001, Mann-Whitney U test). Similarly, the distributions of the two states were significantly different among the normalised high γ amplitude (Fig. 4b) and normalised *SI* values of θ-high γ or α-high γ (Fig. 4c,d).

### Detection of an epileptic seizure

**Figure 5:**
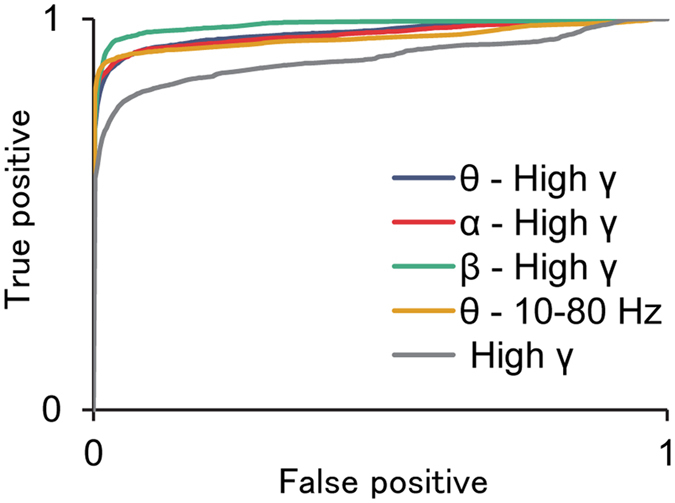
ROC curves to detect seizures using the *SI* values. To differentiate the ictal state from the interictal state, ROC curves were plotted for normalised *SI* values of θ-high γ, α-high γ, and β-high γ, and θ phase for the 10–80 Hz amplitude and the normalised amplitude of the high γ band.

Using the *SI* values, the ictal state was successfully differentiated from the interictal states. The accuracy to classify the ictal state was compared among normalised *SI* values of the θ-high γ, α-high γ, and β-high γ phases of the θ amplitude of 10–80 Hz and the normalised amplitude of the high γ band alone. Among those, the ictal state was classified accurately with the highest specificity and sensitivity by the normalised *SI* values of β-high γ (Fig. 5). The ROC of the normalised *SI* values of β-high γ (AUC, 0.9843) used to classify the ictal state has a significantly larger AUC than others, namely, the normalised *SI* values of θ-high γ (AUC, 0.9656; *p* < 0.000001, see Methods), the normalised *SI* of α-high γ (AUC, 0.9616; *p* < 0.000001), the normalised *SI* of θ-phase 10–80 Hz amplitude (AUC, 0.9525; *p* < 0.000001), and the normalised amplitude of the high γ band (AUC, 0.8996; *p* < 0.000001).

For each feature, the accuracy to detect the seizure was evaluated using the remaining 14 seizures. The normalized *SI* values of β-high γ demonstrated the largest sensitivity among the four types of PAC, with a small FDR < 1 (Table 2). Moreover, FDR smaller than high frequency oscillations with similar sensitivity was also shown.

## Discussion

The present study clearly revealed that iEEG signals during a seizure were successfully characterised by a significantly stronger PAC between the high γ amplitude and lower-frequency phase compared to iEEG of the interictal state in patients with MRI-negative TLE. Among several combinations of frequency bands, PAC between the β-phase and high γ amplitude was shown to classify a seizure of refractory TLE most accurately. Using the *SI* values of high γ amplitude, the ictal state was classified from the 24-h iEEG with a higher sensitivity and specificity compared to detection using the other *SI* values or high γ amplitude alone. These results demonstrated that PAC of iEEG is useful for the detection of epileptic seizures.

Some previous reports demonstrated that PAC characterises seizures and epileptic mechanisms^15^. In addition, it was suggested that the spatial distribution of PAC might be useful to identify a resection area for epilepsy surgery^16^. It has not been elucidated whether PAC can be used to differentiate the ictal state from the interictal state. Similar to the previous studies^22^, our results clearly demonstrate that the SI values during the ictal state were significantly higher than the phase-shuffled SI values of the same signals; however, the significantly high SI values during the ictal state do not necessarily mean that the PAC values during the ictal state is statically stronger than from those during the interictal state. Actually, the SI values between the θ phase and the 10–80 Hz amplitude, and these between β-high γ and θ-high γ were significantly higher than the respective phase-shuffled SI values for the same combination of frequency bands. Moreover these combinations, the SI values between the θ phase and the 10–80 Hz amplitude showed the highest t value. However, the ROC curve showed that seizure classification was significantly more accurate when using the β-high γ PAC compared to the θ phase and 10–80 Hz amplitude PAC or high γ amplitude. Therefore, it is considered that the β-high γ PAC increased more during a seizure than the other types of PACs in patients diagnosed with MRI-negative TLE. These changes in PAC during a seizure potentially enable the successful differentiation of PAC during the ictal state from the physiological PAC during the interictal state, which changes depending on the patient’s behaviour and the location of the contacts in the brain^12^. PAC during the ictal state might be a result of the abnormal neural dynamics generated during seizure, which are different from the exaggerated activation of neural dynamics generating PAC during an interictal state.

We demonstrated that *SI* values are more accurate for the classification of the ictal state compared to high γ amplitude alone. Although previous studies reported the significance of high γ amplitude in seizures^24^,^25^,^26^,^27^, some recent studies revealed that PAC characterises epileptic activity more accurately than high γ amplitude^15^,^28^. Our results support the hypothesis that PAC characterises epileptic activity. Moreover, the significantly high classification accuracy, and the sensitivity and the FDR of seizure detection using the *SI* values suggested the clinical significance of PAC of iEEG for seizure detection compared to the other features of iEEG^17^,^29^,^30^,^31^, scalp EEG^32^,^33^,^34^, and other biological signals^35^,^36^,^37^. Notably, the sensitivity and the FDR using β-high γ PAC were comparable with or even superior to that of previous studies^38^,^39^,^40^, although seizures were detected simply by thresholding the PAC in our algorithm.

It is well known that the characteristic frequency range of phase coupled with high γ amplitude varies depending on anatomical location and seizure type. In the temporal lobe, PAC between the θ phase and high γ amplitude is observed dominantly^11^,^12^. Conversely, PAC between the α phase and high γ amplitude is dominant in the occipital lobe^41^. Therefore, the characteristics of frequency bands might change depending on the location of the epileptogenic lesion. In addition, it is known that the ictal iEEG of epileptic spasms is characterized by brief high γ oscillations coupled with the phase of the slow delta wave (such as 0.3 Hz)^36^. The coupling between the high γ amplitude and the phase of the δ band or the other lower-frequency bands is potentially useful for detecting epileptic spasms or other types of seizures such as tonic seizures^42^. Because PAC between the phase of the β band and the amplitude of the high γ band characterised seizures in MRI-negative TLE patients, as shown in our results, further evaluation of characteristic frequencies in different types of epilepsy in a larger population would determine the clinical utility of our algorithm for the detection of ictal events.

The limitation of this study is the small amount of data, although the significant accuracy we observed in seizure classification in a relatively small patient sample suggests a reasonable effect size. To show the clinical significance of PAC to detect seizures, a prospective study with a larger sample size including patients with various epilepsy syndromes is warranted.

We demonstrated that PAC was able to classify and detect the ictal state among the long-term interictal states with high sensitivity and specificity. However, it should be noted that we did not demonstrate that PAC predicts the ictal state before seizure onset. Another elaborate study will be necessary to develop an algorithm for seizure prediction using PAC.

In conclusion, we have shown that PAC is an electrophysiological biomarker for the detection of the ictal state. Analysis of PAC during ictal and interictal states elucidates the characteristic dynamics of seizures and their relationship to interictal brain activity.

## Additional Information

**How to cite this article**: Edakawa, K. *et al*. Detection of Epileptic Seizures Using Phase–Amplitude Coupling in Intracranial Electroencephalography. *Sci. Rep*. **6**, 25422; doi: 10.1038/srep25422 (2016).

## Supplementary Material

Supplementary Information

## Figures and Tables

**Table 1:** Clinical profiles of the patients.

Patient	Age (y)/Sex	Number of contacts	Electrode depth	Number of seizures	Engel’s classification	Recording time*
A	37/M	83	−	1	III	12 days
B	30/F	113	+	1	I	9 days
C	43/M	50	+	2	II	7 days
D	32/M	113	+	3	III	12 days
E	21/M	90	+	1	III	12 days
F	35/F	85	+	2	I	10 hours
G	24/M	74	+	11	I	12 days

^*^Total time of iEEG signals recorded at 1000–10,000 Hz. F, female; M, male.

**Table 2:** Sensitivity and FDR of seizure detection using PAC.

Feature	Sensitivity (%)	FDR (per hour)
θ-high γ	78.6	0
α-high γ	78.6	0
β-high γ	92.9	0.023
θ phase of 10–80 Hz amplitude	42.9	0
High γ amplitude alone	100	0.713

FDR, false detection rate; PAC, phase–amplitude coupling.

## References

[b1] Burkholder D. B. . Interictal scalp electroencephalography and intraoperative electrocorticography in magnetic resonance imaging-negative temporal lobe epilepsy surgery. JAMA Neurol. 71, 702–709 (2014). 24781216 10.1001/jamaneurol.2014.585 PMC4183227

[b2] Cook M. J. . Prediction of seizure likelihood with a long-term, implanted seizure advisory system in patients with drug-resistant epilepsy: a first-in-man study. Lancet. Neurol. 12, 563–571 (2013). 23642342 10.1016/S1474-4422(13)70075-9

[b3] Meier R., Dittrich H., Schulze-Bonhage A. & Aertsen A. Detecting epileptic seizures in long-term human EEG: a new approach to automatic online and real-time detection and classification of polymorphic seizure patterns. J Clin Neurophysiol 25, 119–131 (2008). 18469727 10.1097/WNP.0b013e3181775993

[b4] Xia Y. . Seizure detection approach using S-transform and singular value decomposition. Epilepsy & behav 52, 187–193 (2015). 10.1016/j.yebeh.2015.07.043 26439656

[b5] Osorio I., Frei M. G., Lozano A. M. & Wennberg R. Subcortical (thalamic) automated seizure detection: A new option for contingent therapy delivery. Epilepsia 56, e156–160 (2015). 26332340 10.1111/epi.13124

[b6] Rosenow F. & Luders H. Presurgical evaluation of epilepsy. Brain 124, 1683–1700 (2001). 11522572 10.1093/brain/124.9.1683

[b7] Fisher R. S. . High-frequency EEG activity at the start of seizures. J Clin Neurophysiol 9, 441–448 (1992). 1517412 10.1097/00004691-199207010-00012

[b8] Alarcon G., Binnie C. D., Elwes R. D. & Polkey C. E. Power spectrum and intracranial EEG patterns at seizure onset in partial epilepsy. Electroencephalogr. Clin. Neurophysiol. 94, 326–337 (1995). 7774519 10.1016/0013-4694(94)00286-t

[b9] Worrell G. A. . High-frequency oscillations and seizure generation in neocortical epilepsy. Brain 127, 1496–1506 (2004). 15155522 10.1093/brain/awh149

[b10] Ikeda A. . Focal ictal direct current shifts in human epilepsy as studied by subdural and scalp recording. Brain 122, 827–838 (1999). 10355669 10.1093/brain/122.5.827

[b11] Canolty R. T. . High gamma power is phase-locked to theta oscillations in human neocortex. Science 313, 1626–1628 (2006). 16973878 10.1126/science.1128115 PMC2628289

[b12] Canolty R. T. & Knight R. T. The functional role of cross-frequency coupling. Trends Cogn. Sci. 14, 506–515 (2010). 20932795 10.1016/j.tics.2010.09.001 PMC3359652

[b13] Axmacher N. . Cross-frequency coupling supports multi-item working memory in the human hippocampus. Proc Natl Acad Sci USA 107, 3228–3233 (2010). 20133762 10.1073/pnas.0911531107 PMC2840289

[b14] Yanagisawa T. . Regulation of motor representation by phase-amplitude coupling in the sensorimotor cortex. J. Neurosci. 32, 15467–15475 (2012). 23115184 10.1523/JNEUROSCI.2929-12.2012 PMC6621589

[b15] Weiss S. A. . Ictal high frequency oscillations distinguish two types of seizure territories in humans. Brain 136, 3796–3808 (2013). 24176977 10.1093/brain/awt276 PMC3859220

[b16] Weiss S. A. . Seizure localization using ictal phase-locked high gamma: A retrospective surgical outcome study. Neurology 84, 2320–2328 (2015). 25972493 10.1212/WNL.0000000000001656 PMC4464742

[b17] Le Van Quyen M. . Spatio-temporal characterizations of non-linear changes in intracranial activities prior to human temporal lobe seizures. Eur J Neurosci 12, 2124–2134 (2000). 10886352 10.1046/j.1460-9568.2000.00088.x

[b18] Asano E. . Role of subdural electrocorticography in prediction of long-term seizure outcome in epilepsy surgery. Brain 132, 1038–1047 (2009). 19286694 10.1093/brain/awp025 PMC2668945

[b19] Zijlmans M. . Ictal and interictal high frequency oscillations in patients with focal epilepsy. Clin Neurophysiol 122, 664–671 (2011). 21030302 10.1016/j.clinph.2010.09.021 PMC3771929

[b20] Gotman J., Levtova V. & Farine B. Graphic representation of the EEG during epileptic seizures. Electroencephalogr. Clin. Neurophysiol. 87, 206–214 (1993). 7691551 10.1016/0013-4694(93)90020-v

[b21] Asano E. . Quantitative interictal subdural EEG analyses in children with neocortical epilepsy. Epilepsia 44, 425–434 (2003). 12614399 10.1046/j.1528-1157.2003.38902.x

[b22] Cohen M. X. Assessing transient cross-frequency coupling in EEG data. J Neurosci Methods 168, 494–499 (2008). 18061683 10.1016/j.jneumeth.2007.10.012

[b23] Hanley J. A. & McNeil B. J. The meaning and use of the area under a receiver operating characteristic (ROC) curve. Radiology 143, 29–36 (1982). 7063747 10.1148/radiology.143.1.7063747

[b24] Jacobs J. . High-frequency oscillations (HFOs) in clinical epilepsy. Prog. Neurobiol. 98, 302–315 (2012). 22480752 10.1016/j.pneurobio.2012.03.001 PMC3674884

[b25] Holler Y. . High-frequency oscillations in epilepsy and surgical outcome. A meta-analysis. Front. Hum. Neurosci. 9, 574 (2015). 26539097 10.3389/fnhum.2015.00574 PMC4611152

[b26] Ochi A. . Dynamic changes of ictal high-frequency oscillations in neocortical epilepsy: using multiple band frequency analysis. Epilepsia 48, 286–296 (2007). 17295622 10.1111/j.1528-1167.2007.00923.x

[b27] Dumpelmann M., Jacobs J. & Schulze-Bonhage A. Temporal and spatial characteristics of high frequency oscillations as a new biomarker in epilepsy. Epilepsia 56, 197–206 (2015). 25556401 10.1111/epi.12844

[b28] Ibrahim G. M. . Dynamic modulation of epileptic high frequency oscillations by the phase of slower cortical rhythms. Exp. Neurol. 251, 30–38 (2014). 24211781 10.1016/j.expneurol.2013.10.019

[b29] Qu H. & Gotman J. A seizure warning system for long-term epilepsy monitoring. Neurology 45, 2250–2254 (1995). 8848202 10.1212/wnl.45.12.2250

[b30] Le Van Quyen M. . Anticipation of epileptic seizures from standard EEG recordings. Lancet. 357, 183–188 (2001). 11213095 10.1016/S0140-6736(00)03591-1

[b31] Osorio I. . Performance reassessment of a real-time seizure-detection algorithm on long ECoG series. Epilepsia 43, 1522–1535 (2002). 12460255 10.1046/j.1528-1157.2002.11102.x

[b32] Gotman J. Automatic recognition of epileptic seizures in the EEG. Electroencephalogr. Clin. Neurophysiol. 54, 530–540 (1982). 6181976 10.1016/0013-4694(82)90038-4

[b33] Pauri F., Pierelli F., Chatrian G. E. & Erdly W. W. Long-term EEG-video-audio monitoring: computer detection of focal EEG seizure patterns. Electroencephalogr. Clin. Neurophysiol. 82, 1–9 (1992). 1370137 10.1016/0013-4694(92)90175-h

[b34] Saab M. E. & Gotman J. A system to detect the onset of epileptic seizures in scalp EEG. Clin. Neurophysiol. 116, 427–442 (2005). 15661120 10.1016/j.clinph.2004.08.004

[b35] Szabo C. A. . Electromyography-based seizure detector: Preliminary results comparing a generalized tonic-clonic seizure detection algorithm to video-EEG recordings. Epilepsia 56, 1432–1437 (2015). 26190150 10.1111/epi.13083

[b36] Eggleston K. S., Olin B. D. & Fisher R. S. Ictal tachycardia: the head-heart connection. Seizure 23, 496–505 (2014). 24698385 10.1016/j.seizure.2014.02.012

[b37] Varon C., Jansen K., Lagae L. & Van Huffel S. Can ECG monitoring identify seizures? J. Electrocardiol. 48, 1069–1074 (2015). 26324174 10.1016/j.jelectrocard.2015.08.020

[b38] Kuhlmann L. . Seizure detection using seizure probability estimation: comparison of features used to detect seizures. Ann. Biomed. Eng. 37, 2129–2145 (2009). 19590961 10.1007/s10439-009-9755-5

[b39] Zheng Y. X. . An automatic patient-specific seizure onset detection method using intracranial electroencephalography. Neuromodulation 18, 79–84, discussion 84 (2015). 25113135 10.1111/ner.12214

[b40] Ramgopal S. . Seizure detection, seizure prediction, and closed-loop warning systems in epilepsy. Epilepsy Behav. 37, 291–307 (2014). 25174001 10.1016/j.yebeh.2014.06.023

[b41] Osipova D., Hermes D. & Jensen O. Gamma power is phase-locked to posterior alpha activity. PloS. One 3, e3990 (2008). 19098986 10.1371/journal.pone.0003990 PMC2602598

[b42] Guirgis M., Chinvarun Y., Carlen P. L. & Bardakjian B. L. The role of delta-modulated high frequency oscillations in seizure state classification. *Conf. Proc. IEEE. Eng. Med. Biol. Soc*. **2013**, 6595-6598 (2013). 10.1109/EMBC.2013.6611067 24111254

