# Supplementary material for: Detection of Epileptic Seizures Using Phase–Amplitude Coupling in Intracranial Electroencephalography
Source: Sci Rep. 2016 May 5;6:25422. doi: 10.1038/srep25422 (PMC4857088; doi:10.1038/srep25422)
Supplement: Supplementary Information [file srep25422-s1.pdf]

Detection of Epileptic Seizures Using Phase–Amplitude Coupling in Intracranial Electroencephalography

+Kohtaroh Edakawa[1,5], +Takufumi Yanagisawa[1-5], Haruhiko Kishima[1,5]*, Ryohei Fukuma[1,3], Satoru Oshino[1,5], Hui Ming Khoo [1,5], Maki Kobayashi[1,5], Masataka Tanaka[1,5], Toshiki Yoshimine[1,5]

[1] Osaka University Graduate School of Medicine, Department of Neurosurgery, Suita 565-0871, Osaka, Japan

[2] Osaka University Graduate School of Medicine, Division of Functional Diagnostic Science, Suita 565-0871, Osaka, Japan

[3] ATR Computational Neuroscience Laboratories, Department of Neuroinformatics, Seika-cho 619-0288, Kyoto, Japan

[4] JST PRESTO, Suita 565-0871, Osaka, Japan

[5] Osaka University Hospital Epilepsy Center, Suita 565-0871, Osaka, Japan

* Correspondence to: hkishima@nsurg.med.osaka-u.ac.jp
[+] These authors contributed equally to this work.

SUPPLEMENTARY MATERIAL

Supplementary Figure

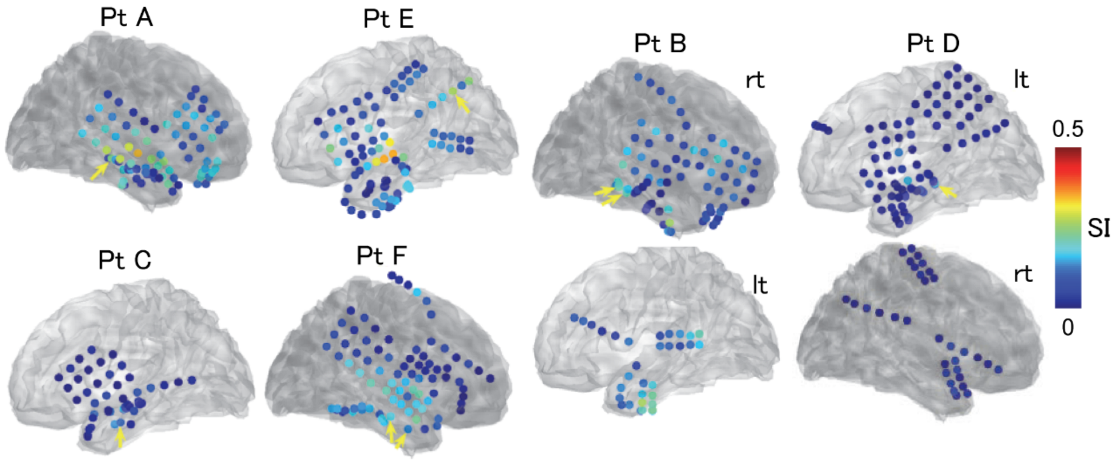

Supplementary Figure S1. The averaged *SI* of β–high γ values during the ictal state was colour-coded at the location of each contact on a normalised brain in all of the other patients. Patients B and D had implants on both sides (rt: right side; lt: left side). The yellow arrowhead indicates the epileptogenic zone.

Supplementary Methods

To examine whether the threshold for the normalized *SI* value was generalised among patients, we evaluated the accuracy detecting the ictal state using a leave-one-subject-out cross-validation (LOOCV) procedure[23]. First, the ROC curve was plotted as mentioned using the data sets of six of the seven patients (training data set) and leaving out the data set of one patient (test data set). Then, using the ROC curve, we selected a point that was nearest to the point of perfect classification (false positive, true positive = 0, 1). The threshold used to plot this point was determined as an optimal threshold value to distinguish the ictal state from the interictal state[24]. The optimal threshold value was then applied to the data set of the patient whose data were left out to test how accurately the ictal state could be distinguished from the interictal state of this patient. The criterion to detect the ictal state was the same as that of the ROC analysis. The same procedure was repeated for seven rounds by leaving out the data set of a different patient in each round. The sensitivity and specificity of the detection were averaged among seven patients for each of the features. One-way ANOVA was performed to test the differences of the sensitivity and specificity among features.

Supplementary Result

The classification accuracy was validated by the LOOCV procedure. The cross-validation showed that PAC successfully differentiated the ictal state from the interictal state with high accuracy (Supplementary Table S1).

Supplementary Discussion

The LOOCV procedure has a risk of overestimation. The independent data set might be necessary for better estimation. However, our study showed that the distributions of SI values were significantly different enough between the ictal state and the interictal state to be successfully classified. The successful classification suggests that the SI values are useful to detect the ictal state among the interictal state.

Supplementary Table S1. The cross-validation of PAC distinguished the ictal state from the interictal state

Feature	Sensitivity (mean ± SD)	Specificity (mean ± SD)
θ–high γ	90.5 ± 9.2	93.4 ± 2.8
α–high γ	90.1 ± 7.7	93.6 ± 4.5
β–high γ	94.3 ± 5.9	96.5 ± 3.1
θ phase of 10–80 Hz amplitude	90.1 ± 8.4	95.6 ± 2.4
High γ amplitude alone	80.5 ± 29.0	89.8 ± 8.1

p = 0.508 and 0.096 for each state. One-way ANOVA. n = 5.
